# Supplementary material for: Association between Tooth Loss and Alzheimer’s Disease in a Nested Case–Control Study Based on a National Health Screening Cohort
Source: J Clin Med. 2021 Aug 24;10(17):3763. doi: 10.3390/jcm10173763 (PMC8432055; doi:10.3390/jcm10173763)
Supplement: Supplementary file 1 [file jcm-10-03763-s001.zip › jcm-1334307-supplementary.pdf]

Table S1. Subgroup analyses of odds ratios (95% confidence intervals) of total teeth loss for Alzheimer's disease (per 16 1oss of teeth) according to obesity, smoking, alcohol consumption, blood pressure, fasting blood glucose, and total cholesterol.

**Characteristics**	**Odds ratios for AD**			
	**Crude**	***p* value**	**Adjusted**[2]	***p* value**
BMI <23 (n = 16,554)				
Total teeth loss	1.14 (1.04-1.25)	0.005[1]	1.10 (1.00-1.20)	0.046[1]
BMI ≥23 (n = 23,256)				
Total teeth loss	1.19 (1.08-1.31)	<0.001[1]	1.16 (1.05-1.28)	0.004[1]
Nonsmoker (n = 31,316)				
Total teeth loss	1.15 (1.07-1.24)	<0.001[1]	1.10 (1.02-1.19)	0.014[1]
Past smoker and current smoker (n = 8,494)				
Total teeth loss	1.30 (1.13-1.49)	<0.001[1]	1.23 (1.07-1.42)	0.005[1]
Alcohol consumption <1 time a week (n = 29,158)				
Total teeth loss	1.12 (1.04-1.21)	0.002[1]	1.08 (1.00-1.16)	0.055
Alcohol consumption ≥1 time a week (n = 10,652)				
Total teeth loss	1.41 (1.22-1.63)	<0.001[1]	1.35 (1.17-1.57)	<0.001[1]
Systolic blood pressure <140 mmHg and diastolic blood pressure <90 mmHg (n = 27,488)				
Total teeth loss	1.21 (1.12-1.32)	<0.001[1]	1.16 (1.07-1.26)	<0.001[1]
Systolic blood pressure ≥140 mmHg or diastolic blood pressure ≥90 mmHg (n = 12,322)				
Total teeth loss	1.12 (1.00-1.25)	0.054	1.05 (0.94-1.18)	0.373
Fasting blood glucose <100 mg/dL (n = 22,827)				
Total teeth loss	1.18 (1.09-1.29)	<0.001[1]	1.13 (1.04-1.23)	0.006[1]
Fasting blood glucose ≥100 mg/dL (n = 16,983)				
Total teeth loss	1.19 (1.07-1.31)	<0.001[1]	1.11 (1.00-1.23)	0.042[1]
Total cholesterol <200 mg/dL (n = 22,299)				
Total teeth loss	1.15 (1.05-1.25)	0.002[1]	1.10 (1.00-1.20)	0.040[1]
Total cholesterol ≥200 mg/dL (n = 17,511)				
Total teeth loss	1.22 (1.11-1.35)	<0.001[1]	1.16 (1.05-1.28)	0.004[1]

Note: AD-Alzheimer's disease; BMI-body mass index; CCI-Charlson Comorbidity Index.

[1] Logistic regression model. Significance at p <0.05.

[2] Models adjusted for obesity, smoking, alcohol consumption, systolic blood pressure, diastolic blood pressure, fasting blood glucose, total cholesterol, and CCI scores.

Table S2. Odds ratios (95% confidence intervals) of upper teeth loss for Alzheimer's disease (per 16 1oss of teeth) with subgroup analyses according to age and sex, income, and region of residence.

**Characteristics**	**Odds ratios for AD**			
	**Crude**[2]	***p* value**	**Adjusted**[23]	***p* value**
Total participants (n = 39,810)				
Upper teeth loss	1.35 (1.20-1.52)	<0.001[1]	1.27 (1.12-1.43)	<0.001[1]
Age <75 years old, men (n = 7,895)				
Upper teeth loss	2.00 (1.42-2.79)	<0.001[1]	2.00 (1.42-2.79)	<0.001[1]
Age <75 years old, women (n = 10,760)				
Upper teeth loss	1.66 (1.26-2.18)	0.003[1]	1.55 (1.17-2.05)	0.002[1]
Age ≥75 years old, men (n = 8,740)				
Upper teeth loss	1.35 (1.07-1.70)	0.011[1]	1.28 (1.02-1.62)	0.035[1]
Age ≥75 years old, women (n = 12,415)				
Upper teeth loss	1.11 (0.92-1.33)	0.283	1.06 (0.88-1.28)	0.518
Low income, urban (n = 5,720)				
Upper teeth loss	1.13 (0.76-1.69)	0.538	1.04 (0.69-1.56)	0.849
Low income, rural (n = 11,825)				
Upper teeth loss	1.36 (1.13-1.63)	0.001[1]	1.29 (1.07-1.56)	0.009[1]
High income, urban (n = 9,240)				
Upper teeth loss	1.42 (1.05-1.92)	0.024[1]	1.31 (0.96-1.78)	0.087
High income, rural (n = 13,025)				
Upper teeth loss	1.37 (1.12-1.67)	0.003[1]	1.30 (1.06-1.60)	0.012[1]

Note: AD-Alzheimer's disease; CCI-Charlson Comorbidity Index.

[1] Conditional logistic regression model. Significance at p <0.05.

[2] Models stratified by age, sex, income, and region of residence.

[3] Models adjusted for obesity, smoking, alcohol consumption, systolic blood pressure, diastolic blood pressure, fasting blood glucose, total cholesterol, and CCI scores.

Table S3. Odds ratios (95% confidence intervals) of lower teeth loss for Alzheimer's disease (per 16 1oss of teeth) with subgroup analyses according to age and sex, income, and region of residence.

**Characteristics**	**Odds ratios for AD**			
	**Crude**[2]	***p* value**	**Adjusted**[23]	***p* value**
Total participants (n = 39,810)				
Lower teeth loss	1.37 (1.20-1.57)	<0.001[1]	1.29 (1.12-1.47)	<0.001[1]
Age <75 years old, men (n = 7,895)				
Lower teeth loss	1.65 (1.09-2.49)	0.017[1]	1.36 (0.89-2.09)	0.159
Age <75 years old, women (n = 10,760)				
Lower teeth loss	1.55 (1.15-2.10)	0.005[1]	1.47 (1.08-2.01)	0.014[1]
Age ≥75 years old, men (n = 8,740)				
Lower teeth loss	1.59 (1.22-2.07)	<0.001[1]	1.49 (1.14-1.95)	0.004[1]
Age ≥75 years old, women (n = 12,415)				
Lower teeth loss	1.15 (0.94-1.41)	0.163	1.10 (0.90-1.35)	0.359
Low income, urban (n = 5,720)				
Lower teeth loss	1.71 (1.12-2.61)	0.013[1]	1.63 (1.06-2.50)	0.027[1]
Low income, rural (n = 11,825)				
Lower teeth loss	1.31 (1.06-1.61)	0.011[1]	1.24 (1.00-1.53)	0.051
High income, urban (n = 9,240)				
Lower teeth loss	1.31 (0.93-1.86)	0.126	1.14 (0.80-1.63)	0.466
High income, rural (n = 13,025)				
Lower teeth loss	1.39 (1.10-1.75)	0.005[1]	1.34 (1.06-1.69)	0.015[1]

Note: AD-Alzheimer's disease; CCI-Charlson Comorbidity Index.

[1] Conditional logistic regression model. Significance at $p < 0.05$.

[2] Models stratified by age, sex, income, and region of residence.

[3] Models adjusted for obesity, smoking, alcohol consumption, systolic blood pressure, diastolic blood pressure, fasting blood glucose, total cholesterol, and CCI scores.
